# The Sarah evaluation scale for children and adolescents with cerebral palsy: description and results

**DOI:** 10.1590/bjpt-rbf.2014.0156

**Published:** 2016-03-22

**Authors:** Katia S. Pinto, Camila G. C. Carvalho, Lilian Nakamoto, Luiz G. N. Nunes

**Affiliations:** 1 Programa de Reabilitação Infantil, Rede Sarah de Hospitais de Reabilitação, Brasília, DF, Brazil

**Keywords:** rehabilitation, motor skills assessment, functional skills assessment, cerebral palsy

## Abstract

**Background:**

Assessments of motor-functional aspects in cerebral palsy are crucial to rehabilitation programs.

**Objective:**

To introduce the Sarah motor-functional evaluation scale and to report the initial results of its measurement properties. This scale was created based on the experience of the Sarah Network of Rehabilitation Hospitals in the care of children and adolescents with cerebral palsy.

**Method:**

Preliminary results concerning the measurement properties of the scale were obtained via assessment of 76 children and adolescents with cerebral palsy. Experts' opinions were used to determine an expected empirical score by age group and to differentiate severity levels.

**Results:**

The scale exhibited a high Cronbach’s alpha coefficient (0.95). Strong correlation was observed with experts’ classification for severity levels (0.81 to 0.97) and with the scales Gross Motor Function Measure and Pediatric Evaluation of Disability Inventory (0.80 to 0.98). Regression analysis detected a significant relationship between the scale score and the severity of the child’s motor impairment. The inter-rater reliability was also strong (intraclass correlation coefficient ranging from 0.98 to 0.99). The internal responsiveness of the scale score was confirmed by significant differences between longitudinal evaluations (paired Student’s t test with p<0.01; standardized response mean of 0.60).

**Conclusion:**

The Sarah scale provides a valid measure for assessing the motor skills and functional performance of children and adolescents with cerebral palsy. The preliminary results showed that the Sarah scale has potential for use in routine clinical practice and rehabilitation units.

## Bullet Points

- The Sarah motor-functional evaluation scale was based on 45 years of experience in cerebral palsy rehabilitation.
- Although comprehensive in scope, the scale consists of easily understood items.
- Daily rehabilitation activities easily allow the completion of the scale items.
- Preliminary results on the scale's measurement properties are strong.
- The scale has potential for use in clinical practices as well as in scientific research.

## Introduction

A child with cerebral palsy (CP) may have impairments ranging from mildly uncoordinated movements or abnormal gait pattern to inability to hold objects, speak, or even swallow^1^ ^,^ ^2^. Children with CP may have muscle tone abnormalities, imbalance in muscle activity, limited joint mobility, and balance disorders that can hinder the acquisition or recovery of motor skills^3^. During the course of their development, affected children may be able to attain complete or partial independence in their activities of daily living or require assistance from others throughout their entire lives^4^. Assessments of these aspects are crucial to rehabilitation programs aimed at improving quality of life according to the child’s potential and the family’s needs^5^ ^,^ ^6^.

There are several well-known and internationally used tools for the evaluation of motor and functional performance^7^ ^-^ ^14^. In this study, we present a scale that may be an alternative or complement to the currently available scales^15^. The development of this scale has followed a path that is different from (actually, opposite to) the way today’s major scales were developed. It was visionary at the time to try to assess children in an objective manner that was standardized across a network of rehabilitation hospitals. We now evaluate the scale in its present form in terms of validity, reliability, internal consistency, and responsiveness. The fact that the scale was developed from the daily care of patients renders the layout, language, structure, and manner of recording the scale items straightforward and quite practical for rehabilitation professionals. The simple scoring of the scale provides immediately accessible and available information in a format designed to identify the child or adolescent’s levels of motor acquisition and/or functional independence.

The Sarah scale emerged from evaluation protocols applied at the Sarah Network of Rehabilitation Hospitals. Since the early 1970s, more than 40,000 patients with CP have received care at the hospitals of the Sarah Network^16^. The first version of the scale was used to classify patients with traumatic brain injury in a previous study at the Sarah Network^17^. After that, the composition of the scale items was assembled, analyzed, and reassembled by the Sarah Network’s rehabilitation team of physical therapists, occupational therapists, and developmental pediatricians (with three to 32 years of experience in the treatment of patients with CP). In order to accomplish this task, consecutive pilot tests were conducted and the professionals interacted in successive meetings to review the results of the pilot applications. Twenty-four versions of the scale were evaluated to produce the present composition.

Upon completion, the Sarah scale consisted of 148 items^18^ ^,^ ^19^ distributed between two dimensions: 1) motor dimension (domains: motor acquisition, 23 items; locomotion, 34 items; gross motor skills, 6 items; and upper limb function, 30 items) and 2) functional dimension (domain: activities of daily living, 55 items). The scale domains are naturally related, but each one emphasizes a particular aspect. The motor acquisition domain focuses on the primary acquisition of skills such as balance, ability to maintain positions (e.g. sit independently), and achieving milestones (e.g. first steps). This domain is important for evaluating the performance of children in their first years of life and for the evaluation of children with severe impairments. The locomotion domain measures the ability and capacity to move from one point to another in space. The gross motor skills domain measures the ability to perform movements that require muscle strength and balance. The upper limb function domain evaluates fine motor skills. The “activities of daily living” domain measures the ability and functional capacity to perform daily tasks and is used to evaluate the patient’s independence from caregivers.

The scores for each of the separate domains and dimensions of the scale, as well as the scale's overall score, can range from 0 to 100 points. Within the domains, the maximum score for each item is equivalent to 100 divided by the number of items in the domain, e.g. each of the 23 items in the motor acquisition domain has a maximum score of 100 divided by 23 points (100/23).

Because of the inherent complexity of its assessment, the locomotion domain is an exception to the scoring criteria described above. The following items are scored out of a maximum of 100/5 points: item 24 (most usual form of locomotion, without assistance from others); item 25 (functional capacity in most frequent form of locomotion); and item 57 (locomotion distance, without assistance from others). The set of items relating to the “Mobility Aids” (items 26 to 56) is scored out of a maximum of 100*(2/5) points. The final score for the locomotion domain is the sum of the scores for all items in this domain and ranges from 0 to 100 points, as in the other domains.

The score for the motor dimension of the Sarah scale is calculated as the arithmetic mean of the points obtained for the domains “motor acquisition”, “locomotion”, “gross motor skills”, and “upper limb function”. The score for the functional dimension consists of the points obtained in its single domain, i.e. “activities of daily living”. The overall score on the scale is the arithmetic mean of the points obtained in the two dimensions, “motor” and “functional”.

A manual was developed to assist the evaluators. This manual contains the necessary instructions for the understanding and classification of each item, including those that require assessing performance quality. A more detailed description of how points are assigned to each item can be also found in the Sarah Scale Manual, available from the website^18^ ^,^ ^19^.

In the following sections, we present the results for the first analysis conducted on the measurement properties of the Sarah scale: discriminant validity and the scale’s internal consistency, concurrent validity, inter-rater reliability, and internal responsiveness.

## Method

### Subjects

The Sarah scale was evaluated for its measurement properties based on the assessment of 76 Brazilian children and adolescents with CP who participated in one of the pediatric rehabilitation programs of the Sarah Network. The parents or legal guardians of each child or adolescent signed an informed consent form allowing them to participate in the study. This study was approved by the Ethics Committee of Associação das Pioneiras Sociais, Brasília, DF, Brazil, according to protocol number CAAE 47512915.6.0000.0022.

A four-level classification for severity^18^ ^,^ ^19^ performed by the Sarah Network’s rehabilitation team found that a high percentage of the subjects was mildly impaired.

### Expected score for age group

One cause of variation in the scores obtained on the Sarah scale is the age range of the subjects. In order to encompass the effect of age on the score, five developmental pediatricians independently assessed the scale items and then established a consensus as to the minimum age at which a child or adolescent without any functional or motor impairment could attain the top score for each item of the scale. Based on these evaluations, an “expected score” for each age group was established for each domain of the scale. Comparison of the actual with the expected score allows analysis of the proportional degree of variability from each of the age groups.

### Concurrent validity

To examine the concurrent validity of the Sarah scale, we compared the scores with those of validated scales that assess similar constructs^20^ – the Gross Motor Function Measure^10^ (GMFM) and the Pediatric Evaluation of Disability Inventory^21^ (PEDI). The GMFM scale has been reported in the literature as an instrument for the assessment of motor development in children with CP^22^ ^,^ ^23^, traumatic injury^24^, Down syndrome^25^, or spinal muscular atrophy^26^. In this study, we used the original version of the GMFM, which has 88 items distributed among the domains lying/rolling, sitting, crawling/kneeling, standing, and walking/running/jumping. The internationally known PEDI scale^27^ ^-^ ^30^ was designed for use in children with a variety of disabilities resulting in functional problems and provides a comprehensive tool for the assessment of development and functional skills capacity as defined by three categories: self-care, mobility, and social function^21^. In this study, we compared the Sarah scale scores with those of the self-care and mobility domains of the PEDI scale.

### Evaluation

The evaluation was performed by six physical therapists and four occupational therapists. They were trained to administer the Sarah scale as well as the GMFM and PEDI scales by direct observation while the subjects were receiving care as part of their rehabilitation programs. When necessary, the evaluations were complemented with information obtained from the family and caregivers.

The Sarah scale and the GMFM and PEDI scales were administered to all subjects during the initial session. In order to test the inter-rater reliability of the Sarah scale, 67 of the 76 subjects underwent two evaluations with this scale. In these cases, all four evaluations (two Sarah, one GMFM, and one PEDI) were performed during the same session but by different evaluators working independently. In a follow-up session at the end of the subjects’ rehabilitation period, mean duration: 7 months and 10 days (SD 27 days; minimum, 5 months 21 days; maximum, 9 months 27 days), 30 of the 76 subjects underwent another evaluation using the Sarah scale to determine its ability to detect changes (internal responsiveness).

A Microsoft Excel spreadsheet was created to generate and store the scores from the evaluations. The evaluator could either use a paper form and then enter the information into the computer or enter the information directly into the spreadsheet.

### Statistical analysis

The discriminant validity of the domains was assessed by analysis of the Spearman’s rank correlation coefficient for the relationship between the examining team’s four-level classification^18^ ^,^ ^19^ and the Sarah Scale score. The domains were further validated using multiple linear regression analysis (controlled for sex and age) to evaluate the effects of the motor impairment type on scoring.

The internal consistency of the scale was analyzed by using Cronbach’s alpha coefficient. According to Bland and Altman^31^, a Cronbach’s alpha ranging between 0.90 and 0.95 is desirable to confirm the internal consistency of a scale for clinical application.

Concurrent validity was tested by comparing the Sarah scale with the GMFM and PEDI scales using Pearson’s product moment correlation coefficient. Intraclass Correlation Coefficient^32^ (ICC) was used to evaluate the inter-rater reliability of the scale by comparing the results of independent evaluations conducted by pairs of examiners. The paired Student’s t test and the Standardized Response Mean^33^ (SRM) were used to assess the ability of the scale to detect changes by comparing the initial evaluation to the follow-up evaluation conducted after the rehabilitation period (mean duration: 7 months).

All data processing was performed with IBM SPSS version 21.0. The results of the inferential tests were considered statistically significant when a two-tailed p-value of <0.05 was obtained.

## Results

### Subjects’ characteristics, expected scores, and time for administration

The most relevant characteristics of the children who participated in this study are presented in Table 1. Our sample of patients with CP had more males than females, with a mean age of 5 years and 6 months (standard deviation, 4 years 1 month; minimum, 1 year 2 months; maximum, 15 years 1 month) and a predominance of diplegia-type motor impairment, followed by quadriplegia and hemiplegia.

**Table 1:** Characteristics of the subjects.

Characteristics	
Number of subjects	76
Sex (female/male)	29/47
Age at first assessment (years)	
Mean (SD)	5.3 (4.1)
Range	1.1 to 15.1
Motor impairment: distribution, n (%)	
Hemiplegia	17 (23)
Diplegia	26 (34)
Triplegia	13 (17)
Quadriplegia	20 (26)
Motor/Functional classification†, n (%)	
Without relevant impairment	1 (1)
Mild impairment	34 (45)
Moderate impairment	17 (23)
Severe impairment	24 (31)

SD: standard deviation; n: number of subjects; %: percentage of the total group.

† Four-level classification^18^ ^,^ ^19^ according to evaluations conducted by staff members of the Sarah Network of Hospitals.

Table 2 shows the details of the “expected score”, established by consensus of developmental pediatricians, for every age group for each of the scale’s domains and dimensions. For example, a child between 3 and 4 years of age with no motor or functional disability is expected to achieve scores of approximately 100 points for the domains of “motor acquisition” and “gross motor skills”, a score of approximately 84 for the “locomotion” domain, a score of approximately 77 for the “upper limb function” domain, total scores of approximately 90 points for the motor dimension and 62 points for the functional dimension (i.e. “activities of daily living” domain), and an overall motor/functional score of approximately 76 points.

**Table 2:** Expected Sarah scale scores for age group†.

Age group	Domain				Dimension		General (Motor/ Functional)
	Motor acquisition	Locomotion gains	Gross motor skills	Upper limb function	Motor	Functional	
<6 months	26.1	0.0	0.0	30.0	14.0	0.0	7.0
6 months – 1 yr	78.3	11.0	0.0	60.0	37.3	3.6	20.5
1–2 years	100.0	72.0	66.7	70.0	77.2	12.7	44.9
2–3 years	100.0	81.7	100.0	73.3	88.8	34.5	61.6
3–4 years	100.0	84.0	100.0	76.7	90.2	61.8	76.0
4–5 years	100.0	100.0	100.0	90.0	97.5	78.2	87.8
5–6 years	100.0	100.0	100.0	96.7	99.2	89.1	94.1
6–7 years	100.0	100.0	100.0	100.0	100.0	98.2	99.1
7–8 years	100.0	100.0	100.0	100.0	100.0	98.2	99.1
>8 years	100.0	100.0	100.0	100.0	100.0	100.0	100.0

† Expected scores for age group for children without any functional or motor impairment, established by developmental pediatricians associated with the Sarah Network of Rehabilitation Hospitals. Each domain ranges from 0-100.

Over the course of the evaluations using the Sarah scale, we observed that less time was required to assess the most extreme cases because the number of items to be evaluated was restricted, whereas in cases of mild impairment the child's performance made it possible to evaluate many items at once. The evaluation also tended to be faster in younger children because the number of items to be evaluated increases with age. Other factors that influenced the evaluation time were the evaluator’s expertise and the quality of the interaction between the evaluator and the patient. The mean time for administration of the scale was 35 minutes, with a standard deviation of 17 minutes.

### Measurement properties of the scale

The first measurement property evaluated for the Sarah scale was its internal consistency. The 76 evaluations of children with CP resulted in a Cronbach’s alpha coefficient of 0.95 for the 148 items of the scale.

The validation of the domains involved analysis of the correlation between the scale scores (in terms of the deviation from the expected score for age group) and the four-level classification performed by the rehabilitation professionals. Table 3 shows that there was strong correlation between the scale and the professionals’ assessment, with coefficients ranging from 0.81 to 0.97 for each domain. In other words, the more severe the motor/functional impairment reported by the rehabilitation professionals, the greater the degree to which the subject’s score deviated from the expected score for his or her age group.

**Table 3:** Comparison of the Sarah scale scores (in terms of the deviation from the expected score for age group) with the four-level evaluations of the rehabilitation professionals.

	Sarah scale vs. four-level classification^†^
	Spearman correlation coefficient (95% CI) n = 76
Motor dimension	0.92 (0.89; 0.95)
Motor acquisition	0.97 (0.95; 0.98)
Locomotion	0.92 (0.88; 0.95)
Gross motor skills	0.94 (0.91; 0.96)
Upper limb function	0.92 (0.87; 0.95)
Functional dimension	0.81 (0.72; 0.88)
Overall motor/functional	0.91 (0.86; 0.94)

CI: confidence interval; n: number of subjects.

† Four-level classification performed by Sarah network staff^18^ ^,^ ^19^.

The domains were further evaluated using a multiple linear regression model to analyze the scores’ sensitivity to the severity of motor impairment. Table 4 shows the regression coefficient for each domain. The regression was adjusted for the effects of sex (0 = male, 1 = female) and age (in years). The type of motor impairment (1 = hemiplegia, 2 = diplegia, 3 = triplegia, and 4 = quadriplegia) showed a significant inverse relationship with all domains and dimensions of the scale. In other words, a more severe motor impairment produced a lower score on the scale. Gender was not significantly associated with the scale scores (p-value, 0.42-0.98), but age was directly and significantly associated with the scale scores (p<0.001). Furthermore, the variables age and type of motor impairment explained most of the variation in the scale scores (R^2^ between 0.58 and 0.80).

**Table 4:** Descriptive statistics and multiple linear regression analysis for each domain of the Sarah scale. The regression evaluated the effects of the distribution of motor impairment on the scale scores and was controlled for sex and age.

n=76	Mean scale score (SD)	Regression beta coefficient (SE)				R^2^
		Intercept	Controlled variable		Type^¥^	
			Sex^†^	Age^‡^		
Motor dimension	53.5 (36.5)	86.6 (8.0)	–2.3 (4.6)	3.5 (0.6)	–20.8 (2.2)	0.73
Motor acquisition	66.6 (38.3)	100.7 (9.0)	–2.5 (5.2)	3.5 (0.7)	–21.3 (2.4)	0.69
Locomotion	44.8 (43.4)	86.8 (11.0)	–1.6 (6.3)	3.5 (0.8)	–24.5 (3.0)	0.65
Gross motor skills	41.2 (40.4)	79.5 (11.2)	–1.6 (6.4)	2.9 (0.8)	–21.8 (3.0)	0.58
Upper limb function	61.5 (32.8)	79.1 (7.5)	–3.5 (4.3)	4.0 (0.5)	–15.6 (2.0)	0.71
Functional dimension	41.4 (37.3)	36.8 (7.1)	2.2 (4.1)	6.3 (0.5)	–12.5 (1.9)	0.80
General motor/functional	47.5 (35.8)	61.2 (7.0)	–0.5 (4.1)	4.9 (0.5)	–16.7 (1.9)	0.79

N: number of subjects; SD: standard deviation; SE: standard error. All regression coefficients were significant (p<0.001) except those for the sex variable, none of which were significant (p>0.420). R^2^: coefficient of determination.

† Sex classification: 0 – male; 1 – female.

‡ Age in years.

¥ Type of motor impairment distribution: 1 – hemiplegia; 2 – diplegia; 3 – triplegia; 4 – quadriplegia.

The scores for the Sarah scale domains/dimensions correlated strongly with those of the GMFM and PEDI scales (correlation coefficients ranging from 0.80 to 0.98; 95% confidence interval ranging from 0.70 to 0.99). As expected, the GMFM motor scale and the mobility dimension of the PEDI scale correlated more strongly with the motor dimension of the Sarah scale, whereas the self-care dimension of the PEDI scale, which is geared more towards the functional evaluation of children, had greater correlation with the functional dimension of the Sarah scale.

Table 5 shows the inter-rater reliability of the scale. The ICC between pairs of independent examiners was strong, ranging from 0.98 to 0.99, for each of the domains and dimensions.

**Table 5:** Comparison of the Sarah scale scores between pairs of independent examiners and between the first and follow-up assessments.

Sarah scale	ICC (95% CI)	Mean difference^†^ (95% CI); SRM
	Examiner 1 vs. Examiner 2	Follow-up minus 1st Evaluation
	(n=67)	(n=30)
Motor dimension	0.998 (0.996; 0.999)	6.6 (2.5; 10.7); 0.60
Motor acquisition	0.995 (0.992; 0.997)	3.7 (0.8; 6.5); 0.48
Locomotion	0.998 (0.996; 0.999)	9.2 (3.1; 15.3); 0.57
Gross motor skills	0.984 (0.973; 0.990)	12.2 (2.6; 21.9); 0.47
Upper limb function	0.992 (0.987; 0.995)	1.3 (–0.6; 3.3); 0.26
Functional dimension	0.996 (0.994; 0.998)	5.4 (1.3; 9.5); 0.49
Overall motor/functional	0.998 (0.997; 0.999)	6.0 (2.3; 9.7); 0.60

ICC: Intraclass Correlation Coefficient, (2, 1) model, absolute agreement type. CI: confidence interval; n: number of subjects; SRM: Standardized Response Mean.

† The difference between the initial evaluation and the follow-up evaluation was significant for all domains and dimensions (p<0.01, paired Student’s t test) except for the upper limb function domain (p=0.173).

We also observed (Table 5) that the scale was able to detect changes, i.e. it produced different scores for the initial and follow-up evaluations, within an average period of seven months. The evaluations differed significantly in all domains and dimensions (paired Student’s t test with p<0.01; and moderate SRM ranging from 0.47 to 0.60) except for the upper limb function domain, which showed a positive but non-significant difference (paired Student’s t test with p=0.173; and small SRM of 0.26).

## Discussion

This study demonstrated that the Sarah scale is a valid and reliable tool for the motor/functional evaluation of children or adolescents with CP. All domain scores correlated strongly with the independent evaluation of the examiners. High Cronbach’s alpha coefficient demonstrated the scale’s internal consistency. The scale also exhibited good level of concurrent validity, with strong correlations between the Sarah scale and the GMFM and PEDI scales, as well as good inter-rater reliability, with high ICCs between the examiners. The domains of the Sarah scale are able to reflect different levels of impairment. Multiple linear regression models controlled for the influences of age and sex were used to confirm the expected correlations between the domain scores and the type of motor impairment.

We confirmed the Sarah scale’s ability to detect changes in children with CP over time. Only the score for the “upper limb function” domain, in the motor dimension of the scale, did not change significantly between the initial and follow-up evaluations. The “upper limb function” domain assesses the ability to perform manual tasks requiring precise motor control such as “puts the paper inside an envelope”, “writes recognizable letters and numbers”, and “uses computer with regular keyboard”. It may be that the duration of follow-up in this study (i.e. seven months) was insufficient to detect significant changes in these tasks. Other items could also be considered for inclusion in this domain and should be explored in future studies.

The independent scoring of each Sarah scale domain can help professionals analyze factors other than motor deficits that might influence a child’s functional performance, such as cognitive, neuropsychological, or sensorial impairments. A score in the functional dimension lower than the score in the motor dimension might indicate that the subject has problems with certain functional activities despite sufficient motor capacity to perform them. Such problems could be caused by cognitive deficits^1^ ^,^ ^5^ ^,^ ^6^ ^,^ ^34^, learning disabilities, or the type of care received. Family habits and daily routines may influence the child's ability to perform self-care activities^35^. Likewise, subjects who score higher on activities of daily living than on motor acquisition have likely developed efficient compensatory strategies that enable greater functional independence despite motor limitations. This possibility is of great clinical relevance, as movement disorders are often accompanied by disturbances of sensation, cognition, communication, perception, and/or behavior, and their functional skills reflect not only the motor potential for performing an action but also the interactions among all of these capacities^3^ ^,^ ^6^ ^,^ ^36^.

### Limitations of the study

From the beginning, the Sarah scale was focused on analyzing domains rather than the isolated significance of each item. The score for each domain was reduced to a simple proportional calculation with a result ranging from 0 to 100. This simplification helps to understand and analyze the results at the domain and dimension levels but may have adverse effects on the mathematical properties and statistical analysis of the scores, such as floor and ceiling effects and/or over- or under-representation of items and domains. These concerns are also valid for traditional scales such as the GMFM and PEDI^37^ ^-^ ^40^. The extent to which these potential problems affect the Sarah scale must be addressed in future studies. Current scoring requires further revision to evaluate the relative “weight” or importance of items and domains. In the scale scoring, the evaluations are added across items to give each person a total score, therefore conducting a Rasch analysis is a plausible option.

The expected score for age group table established in this study allows for the result of the Sarah scale assessment to be expressed as the proportional deviation from the predicted score. It was adopted as an empirical method based on the experience of the Sarah Network’s rehabilitation team. Although the experts' evaluations are expected to be accurate, this simple empirical process should be further validated by a large population-based study.

An empirical four-level evaluation by the experienced professionals of the Sarah Network was used as the reference standard against which to validate the scale domains. This empirical approach was used because each domain of the Sarah scale has particular characteristics that hinder a direct comparison with existing scales. The Gross Motor Function Classification System^41^ (GMFCS), for example, is a five-level scale focused only on motor abilities, such as trunk balance and walking, whereas the Brief Assessment of Motor Function^7^ (BAMF) is a ten-point scale focused on head balance, trunk balance, standing, and walking. Future studies involving further comparisons with these and other scales will be important for exploring the explanatory potential of the Sarah scale domains.

Another issue to be considered is that the scale has been both developed and tested at several of the Sarah Network hospitals with the participation of its staff. Although this first study was performed in collaboration with experienced professionals and under careful control, future examination in multi-institutional and international research would certainly reinforce and extend the results obtained thus far.

## Conclusion

The findings of this study confirm the suitability of the Sarah scale for evaluating the motor/functional performance of children and adolescents with CP. With further analysis and subsequent improvements, the scale has great potential for use in scientific research and health care as it can provide valuable information to support the decisions involved in the rehabilitation process.
